# Plasma Circulating Vitamin C Levels and Risk of Endometrial Cancer: A Bi-Directional Mendelian Randomization Analysis

**DOI:** 10.3389/fmed.2022.792008

**Published:** 2022-03-23

**Authors:** Haoxin Peng, Xiangrong Wu, Yaokai Wen

**Affiliations:** ^1^Nanshan School, Guangzhou Medical University, Guangzhou, China; ^2^The First Affiliated Hospital of Guangzhou Medical University, Guangzhou, China; ^3^Department of Medical Oncology, School of Medicine, Tongji University, Shanghai, China; ^4^Department of Medical Oncology, Shanghai Pulmonary Hospital, Tongji University Medical School Cancer Institute, Tongji University School of Medicine, Shanghai, China

**Keywords:** vitamin C, endometrial cancer, Mendelian randomization, single nucleotide polymorphism, causality

## Abstract

**Background:**

Observational studies indicated that circulating vitamin C (VitC) levels may be correlated with the risk of endometrial cancer (EC). However, the causal effects and direction between them were still unclear.

**Methods:**

In this study, 11 single nucleotide polymorphisms (SNPs) robustly correlated with plasma VitC levels were extracted from the latest genome-wide association study (GWAS), containing 52,018 individuals. Genetic data of EC were obtained from the Endometrial Cancer Association Consortium (ECAC) (12,906 cases and 108,979 controls). An inverse-variance weighted method was utilized as the primary analysis of Mendelian randomization (MR), supplemented by the weighted median, MR Pleiotropy Residual Sum and Outlier test (MR-PRESSO), and MR-Egger methods. Additional sensitivity analyses excluding 3 SNPs with secondary phenotypes were conducted to rule out the possible pleiotropic effects. Potential impacts of several risk factors of EC, such as obesity, body mass index (BMI), hypertension, and diabetes on VitC levels, were assessed. We additionally evaluated the effects of VitC on LDL cholesterol levels, HDL cholesterol levels, and triglycerides levels to probe into the possible mediators in the VitC-EC pathway.

**Results:**

Genetically predicted higher plasma VitC levels (per 1 SD increase, approximately 20 μmol/L) were causally associated with an increased risk of EC overall [odds ratio (*OR*) 1.374, 95% *CI* 1.128–1.674, *p* = 0.0016], supported by complementary sensitivity analyses. In the subgroup analyses, genetically predicted higher levels of VitC were associated with a tendency of increased risks of both endometrioid (*OR*_SD_ 1.324, 95% *CI* 0.959–1.829, *p* = 0.0881) and non-endometrioid histology (*OR*_SD_ 1.392, 95% *CI* 0.873–2.220, *p* = 0.1647) while without statistical significance. The association remained significant after the exclusion of the three pleiotropic SNPs (*OR*_SD_ 1.394, 95% *CI* 1.090–1.784, *p* = 0.0082). The confounders and mediators were unlikely to affect the VitC-EC relationship. The causal effect of EC on VitC levels was not supported (*OR* 1.001, 95% *CI* 0.998–1.004, *p* = 0.4468).

**Conclusions:**

This bi-directional MR study demonstrated a causal risk role of higher circulating VitC at physiological levels on an increased risk of EC, which was independent of confounders and mediators. Further studies are warranted to elucidate the possible mechanisms.

## Introduction

Endometrial cancer (EC), which mainly affects postmenopausal women, is one of the commonest gynecological cancers worldwide (1). During the past few decades, the mortality rate of EC has been rising continuously in developed countries (2). Recently, vitamin C (VitC) has induced great attention as its potentially preventive effect on cancer. VitC is the most important water-soluble antioxidant in dietary fruit and vegetable sources, which may prevent cancer by reducing oxidative DNA damage, such as DNA mutations (3). Previous studies have indicated that VitC at pharmacological levels (i.e., intravenous medication) rather than physiological concentrations (i.e., oral administration) may manifest protective effects of cancers, while the findings were inconsistent. In general, there is relatively strong evidence supporting inverse associations between intake of VitC and breast cancer (4), lung cancer (5), and colon cancer (6). However, in the field of EC, the reports were relatively limited, and the results were inconsistent. The latest meta-analysis comprised of 9 case-control studies and 1 cohort study by Bandera et al. showed that intake of VitC from food resources rather than supplements decreased the risk of EC by 15% (7).

However, given the methodological limitations of observational reports, the effects of confounders, such as body mass index (BMI) and smoking, which were also common risk factors of cancers, cannot be thoroughly evaluated or eliminated. In addition, given that oxidative stress induced by cancer may enhance the consumption of VitC as well, findings were prone to reverse causality (8). Several randomized controlled trials (RCTs) focusing on this issue did not support the protective role of VitC in the development of cancers, whereas the sample size of incident cancer cases was limited (9, 10). Simultaneously, RCTs investigating the effect of VitC intake and EC risk are time-consuming and expensive, largely infeasible in a primary prevention condition. Hence, we may not establish the causal inference based on existing evidence with confidence. Considering EC has caused enormous health burdens worldwide, determining whether intake of VitC authentically plays a role in preventing EC development is important.

Mendelian randomization (MR), using genetic variations as instrumental variables (IVs), is a novel method for causal inference between exposures and outcomes. The genetic variants, utilized as proxies of exposures, are independent of environmental risk factors and determined prior to the diseases, avoiding the influences of confounders and reverse causation (11). It has been reported that single nucleotide polymorphisms (SNPs) can explain around 1.87% of the variance in plasma circulating VitC levels, suggesting that MR can provide a means for evaluating the causality between VitC and EC risk (12). In the present study, we performed a bi-directional MR method to probe into the putative effects of circulating VitC at physiological levels and the risk of EC. Previous MR studies have reported some of the risk factors of EC. For instance, Kho et al. found that higher LDL cholesterol levels were associated with a lower risk of EC overall, while higher HDL cholesterol levels raised the risk of non-endometrioid EC by 20% (13). Higher serum 17β-estradiol levels [odds ratio (OR) 1.09, 95% *CI* 1.06–1.11, *p* < 0.05] and fasting insulin levels (*OR* 2.34, 95% *CI* 1.06–5.14, *p* = 0.03) were also associated with a higher risk of EC (14, 15). Moreover, Nead et al. reported that increased age at menarche decreased the risk of EC by 22% (16). Therefore, we conducted additional MR analyses on these risk factors, which might play a role as a confounder and/or mediator on the VitC-EC pathway (17).

## Materials and Methods

### Genetic Variants Associated With Circulating VitC Levels

Initially, 11 SNPs robustly associated with plasma circulating VitC (i.e., at statistical significance threshold *p* < 5 × 10^−8^) were extracted from the latest study by Zheng et al. (Supplementary Table 1), containing 52,018 individuals of European origin (Table 1) (12). These SNPs aggregately accounted for 1.87% of the variance in VitC levels (12). Besides, all SNPs were kept for further analyses due to the absence of linkage disequilibrium (*r*^2^ < 0.01). Finally, we established the IVs based on these 11 VitC-related SNPs. Subsequently, we manually checked for the secondary phenotypes of each SNP in the PhenoScanner (http://www.phenoscanner.medschl.cam.ac.uk). Three potential pleiotropic SNPs, including rs56738967 with thyroid function, rs9895661 with serum urate, and rs174547 with fatty acids, were identified (Supplementary Table 1). The statistical power was generated using an online platform, “mRnd: Power calculations for Mendelian Randomization” (https://shiny.cnsgenomics.com/mRnd/), which was performed according to the formula derived by Brion et al. (18). On this basis, the effect size (*OR* = 0.85) of VitC on the risk of EC from the latest meta-analysis by Bandera et al. (7) was applied for power calculation at the threshold of the significance level of 0.05.

**Table 1:** Details of traits used in Mendelian randomization analyses.

**Trait**	**Consortium**	**Number of cases/controls**	**Population**	**Pubmed ID**
Plasma vitamin C	Multiple	52,018	European	33203707
Endometrial cancer	ECAC	12,906/ 108,979	European	30093612
Endometrioid histology		8,758/46,126		
Non- endometrioid histology		1,230/35,447		
Hypertension	MRC-IEU	119,731/343, 202	European	NA
Type 1 diabetes	NA	6,683/12,173	European	25751624
Type 2 diabetes	NA	77,418/356, 122	European	32499647
Obesity class 1 (BMI: 30–34.9 kg/m^2^)	GIANT	98,697	European	NA
Obesity class 2 (BMI: 35–39.9 kg/m^2^)	GIANT	72,546	European	NA
Obesity class 3 (BMI: ≥40 kg/m^2^)	GIANT	50,364	European	NA
Body mass index	MRC-IEU	461,460	European	NA
LDL cholesterol levels	UK Biobank	440,546	European	32203549
HDL cholesterol levels		403,943	European	
Triglycerides		441,016	European	

*MRC-IEU, MRC Integrative Epidemiology Unit; GIANT, Genetic Investigation of ANthropometric Traits; ECAC, Endometrial Cancer Association Consortium*.

### Study Participants of Endometrial Cancer

The Endometrial Cancer Association Consortium (ECAC) is a consortium formed to pool EC genetic studies to conduct large-scale genome-wide association study (GWAS) meta-analyses and identify genes associated with EC. Genetic data of EC that derived from European ancestry were obtained from the ECAC (12,906 EC cases and 108,979 controls) (https://ecac-studies.org/) (Table 1), which were publicly available on the MR-Base platform (https://www.mrbase.org/) (19). Subgroup analyses of different histological subtypes of EC, including endometrioid (8,758 cases and 46,126 controls) and non-endometrioid histology (1,230 cases and 35,447 controls) in ECAC, were also implemented.

### Statistical Analysis

Mendelian randomization was applied as our statistical analysis tool, which is strictly subjected to three assumptions (20): (i) the IVs are robustly associated with increased VitC concentrations; (ii) the IVs affect EC only through their effects on increased VitC concentrations directly, and (iii) the IVs are independent of any confounders. Since the SNPs we chose were selected at the genome-wide significance threshold of *p* < 5 × 10^−8^ and the statistical power was 100% (>80%) as evaluated by an online tool (18), the first assumption was satisfied. Weighted median and MR-Egger methods were performed to test the second assumption indirectly. Regarding the sensitivity analysis, potential horizontal pleiotropic effects were obtained based on the intercept of the MR-Egger analyses. The MR Pleiotropy Residual Sum and Outlier (MR-PRESSO) test was applied for identifying potential horizontal pleiotropy and removing outliner variants (21). The heterogeneity test was implemented as well, and *I*^2^ > 50.0% was considered significant. A leave-one-out analysis was conducted to appraise whether the estimation of MR was determined or biased by a single SNP. Single MR analysis was utilized to assess the effect size of individual SNP.

**Figure 1:**
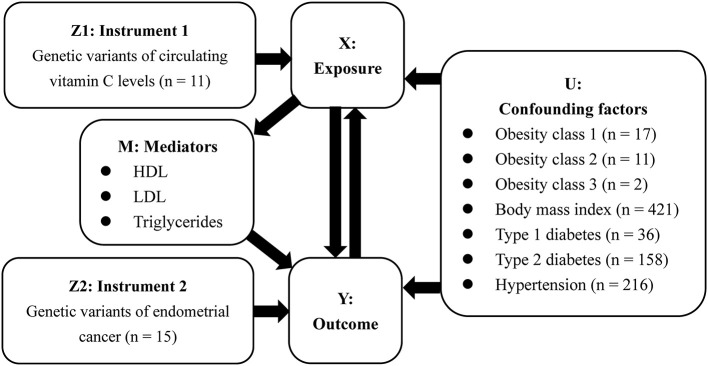
Framework of bi-directional Mendelian randomization analysis in our study.

In this study, we used the random-effects inverse-variance weighted (IVW) to obtain the MR estimate based on multiple IVs. As described in Figure 1, the results were presented as *OR* and 95% *CI*, providing an estimate of relative risk on EC (Y) caused by a per 1 standard deviation (SD) increase in VitC levels (X). Additional sensitivity analysis excluding 3 SNPs with secondary phenotypes was also performed to eliminate the possible pleiotropic effects. In addition, a bi-directional MR analysis was further performed to investigate whether EC (Y) would reversely affect VitC concentrations (X).

Aiming to assess the potential confounders intervening the mechanisms on the VitC-EC relationship, additional MR analyses were performed to investigate whether genetic predisposition toward the common risk factors of EC (U) could be associated with circulating VitC levels. In 2019, Raglan et al. proposed an umbrella review to summarize the risk factors of EC based on literature, in which obesity (strong evidence), diabetes (highly suggestively evidence), and hypertension (suggestively evidence) were stated (17). Additionally, there is evidence that a higher BMI can increase the risk of EC (22). Therefore, further investigation was needed to appraise whether these factors would bias the MR results, leading to the hypothesis of the above-mentioned third assumption. Genetic summary data on obesity were extracted from the GIANT, BMI, and hypertension from the MRC-IEU, and diabetes (both type 1 and type 2) from published GWASs (Table 1). Given that several RCT showed VitC could affect LDL cholesterol levels, HDL cholesterol levels, and triglycerides levels and MR study suggested these factors were associated with the risk of EC, they were considered mediators (M) in the VitC-EC pathway (13, 23). For exposure (i.e., VitC)-mediator analysis, genetic data of these mediators were from the UK Biobank. MR analyses were performed in R (version 4.0.0) using the package TwoSampleMR (version 0.5.1) (24).

## Results

**Figure 2:**
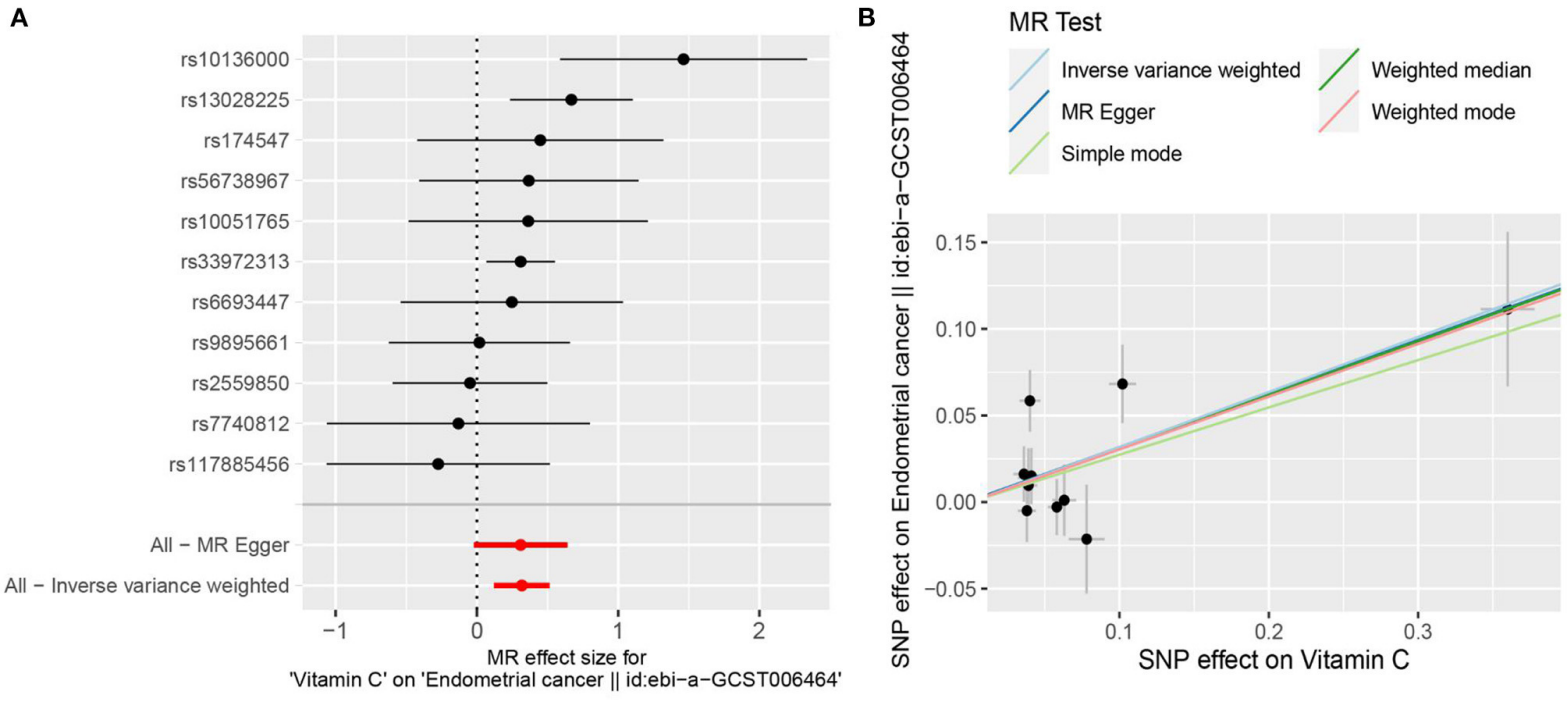
Mendelian randomization estimating the effects of genetically predicted higher plasma circulating vitamin C (VitC) levels (per 1 SD increase, ~20 μmol/L) on the risk of endometrial cancer (EC). **(A)** Forest plot of single nucleotide polymorphisms (SNPs) associated with higher circulating VitC levels and their risk of EC. **(B)** Scatter plots of SNPs correlated with higher circulating VitC concentrations and their risk of EC.

**Figure 3:**
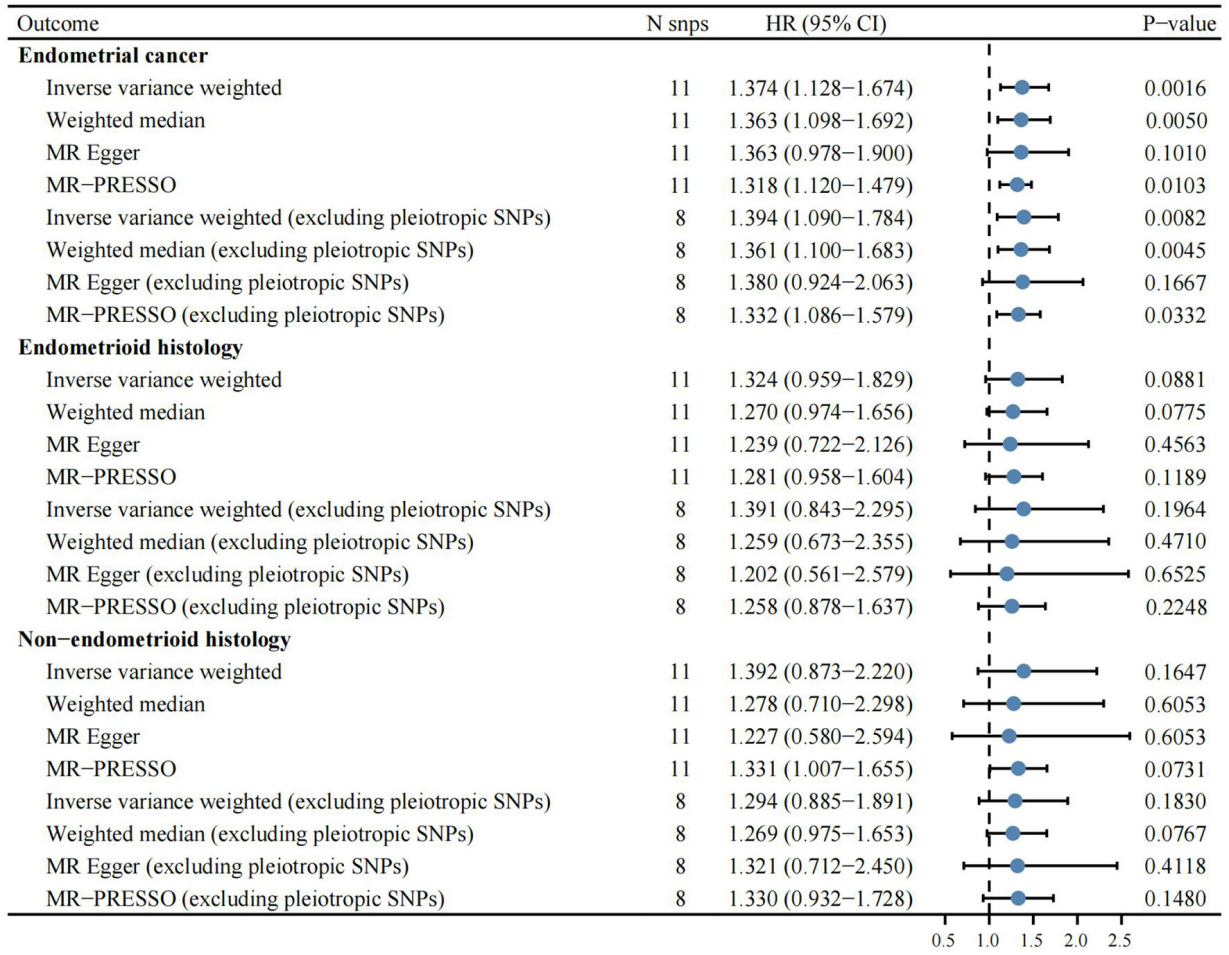
Complementary Mendelian randomization analyses estimating the effects of genetically predicted higher circulating vitamin C levels (per 1 SD increase, ~20 μmol/L) on the risk of endometrial cancer overall and different histological subtypes.

Based on the previously reported effect size (*OR* = 0.85) to evaluate the causal effect of VitC on EC (7), our MR analyses with large-scale consortium had sufficient power (100%). The primary MR results indicated that genetically predisposed higher plasma VitC levels (per 1 SD increase, ~20 μmol/L) were causally associated with an increased EC risk (*OR* 1.374; 95% *CI*, 1.128–1.674; *p* = 0.0016), supported by complementary sensitivity analyses (*OR*_SD_ 1.363, 95% *CI* 1.098–1.692, *p* = 0.0050 for weighted median; *OR*_SD_ 1.318, 95% *CI* 1.120–1.479, *p* = 0.0103 for MR-PRESSO; *OR*_SD_ 1.363, 95% *CI* 0.978–1.900, *p* = 0.1010 for MR-Egger) (Figures 2, 3, Supplementary Table 2). In the subgroup analyses, genetically predicted higher levels of VitC were associated with a tendency of increased risks of both endometrioid (*OR*_SD_ 1.324, 95% *CI* 0.959–1.829, *p* = 0.0881 for IVW; *OR*_SD_ 1.270, 95% *CI* 0.974–1.656, *p* = 0.0775 for weighted median; *OR*_SD_ 1.239, 95% *CI* 0.722–2.216, *p* = 0.4563 for MR-Egger; *OR*_SD_ 1.281, 95% *CI* 0.958–1.604, *p* = 0.1189 for MR-PRESSO) and non-endometrioid histology (*OR*_SD_ 1.392, 95% *CI* 0.873–2.220, *p* = 0.1647 for IVW; *OR*_SD_ 1.278, 95% *CI* 0.710–2.298, *p* = 0.6053 for weighted median; *OR*_SD_ 1.227, 95% *CI* 0.580–2.594, *p* = 0.6053 for MR-Egger; *OR*_SD_ 1.331, 95% *CI* 1.007–1.655, *p* = 0.0731 for MR-PRESSO) while without statistical significance (Figure 3).

After the exclusion of the three pleiotropic SNPs, the association remained significant (*OR*_SD_ 1.394, 95% *CI* 1.090–1.784, *p* = 0.0082 for IVW; *OR*_SD_ 1.361, 95% *CI* 1.100–1.683, *p* = 0.0045 for weighted median; *OR*_SD_ 1.332, 95% *CI* 1.086–1.579, *p* = 0.0322 for MR-PRESSO; *OR*_SD_ 1.380, 95% *CI* 0.924–2.063, *p* = 0.1667 for MR-Egger) (Supplementary Table 3). Despite without statistical significance, the effects of VitC on EC remained directionally consistent in the subgroup analysis (*OR*_SD_ 1.391, 95% *CI* 0.843–2.295, *p* = 0.1964 for endometrioid histology; *OR*_SD_ 1.294, 95% *CI* 0.885–1.891, *p* = 0.1830 for non-endometrioid histology) (Figure 3). Single MR analysis demonstrated that rs10136000 (β = 1.46, *p* = 0.0010, nearest gene-serine-threonine protein kinase), rs13028225 (β = 0.67, *p* = 0.0026, nearest gene-sodium-dependent vitamin C transporter (SVCT) 3), and rs33972313 (β = 0.31, *p* = 0.0124, nearest gene-SVCT 1) were independently associated with an increased EC risk while other SNPs showed insignificant associations with EC (Supplementary Table 4).

### Sensitivity Analysis

Using MR-Egger regression, we further tested for the global pleiotropic effect, wherein no global violation of pleiotropic assumptions existed (intercept = 0.0007, *p* = 0.9516 for EC overall; intercept = 0.0058, *p* = 0.7636 for endometrioid histology; intercept = 0.0110, *p* = 0.6822 for non-endometrioid histology) (Supplementary Table 5). Besides, heterogeneity was not observed (*I*^2^ = 32.65% for EC overall; *I*^2^ = 3.51% for endometrioid histology; *I*^2^ = 20.78% for non-endometrioid histology) in the study (Supplementary Table 6). Leave-one-out studies supported no evidence that a single SNP had an impact on the gross effect of genetically predisposed VitC on EC (Supplementary Table 7). We further evaluated whether the association between genetically predisposed one-SD increase in the VitC concentrations and EC was influenced by potential confounders (i.e., obesity, BMI, diabetes, and hypertension). The IVW results demonstrated that genetically predisposed obesity class 3 (BMI: ≥40 kg/m^2^) (*OR* 0.997, 95% *CI* 0.994–1.000, *p* = 0.0302) and BMI (*OR* 0.991, 95% *CI* 0.987–0.996, *p* < 0.001) but not obesity class 1 (BMI: 30–34.9 kg/m^2^) (*OR* 0.996, 95% *CI* 0.992–1.000, *p* = 0.0789) or obesity class 2 (*OR* 0.998, 95% *CI* 0.995–1.002, *p* = 0.3811) were inversely associated with VitC levels (Table 2, Supplementary Table 8). Genetically predicted hypertension was negatively correlated with VitC levels (*OR* 0.985, 95% *CI* 0.972–0.999, *p* = 0.0306) (Table 2). No causality between diabetes (both type 1 and type 2) and VitC levels was observed (Table 2, Supplementary Table 8). Since the effect size was marginal, the causal direction and association were unlikely to be affected by these confounders.

**Table 2:** Causal effects between genetic predisposition toward common risk factors of endometrial cancer and circulating plasma vitamin C.

**Outcomes**	**Causal effect (95% CI)**	***P*-value**
Obesity class 1 (BMI: 30–34.9 kg/m^2^)	0.996 (0.992, 1.000)	0.0789
Obesity class 2 (BMI: 35–39.9 kg/m^2^)	0.998 (0.995, 1.002)	0.3811
Obesity class 3 (BMI: ≥40 kg/m^2^)	0.997 (0.994, 1.000)	0.0302
Body mass index	0.991 (0.987, 0.996)	<0.001
Type 1 diabetes	1.000 (0.998, 1.001)	0.8007
Type 2 diabetes	1.000 (0.998, 1.002)	0.9312
Hypertension	0.985 (0.972, 0.999)	0.0306

For exposure (i.e., VitC)-mediator effects, consistent with the meta-analysis by McRae et al. that VitC supplementation can decrease LDL cholesterol levels (23), we found that genetic predisposition towards higher circulating VitC levels were associated with a tendency of lower LDL cholesterol levels (*OR* 0.968, 95% *CI* 0.873–1.073, *p* = 0.5385) while without statistical significance. On the contrary, since VitC supplementation was reported to raise HDL cholesterol levels and decrease triglycerides levels (23), we found that genetically predicted higher circulating VitC levels were correlated with lower HDL cholesterol levels (*OR* 0.943, 95% *CI* 0.792–1.122, *p* = 0.5081) and higher triglycerides levels (*OR* 1.062, 95% *CI* 0.908–1.242, *p* = 0.4546) while without statistical significance (Table 3, Supplementary Table 9). Consequently, these mediators appeared to have no bearing on the VitC-EC relation. The causal effect of EC on VitC levels was not supported (*OR* 1.001, 95% *CI* 0.998–1.004, *p* = 0.4468) (Supplementary Table 10).

**Table 3:** Causal effects between genetic predisposition toward circulating plasma vitamin C and mediators.

**Outcomes**	**Causal effect (95% CI)**	***P*-value**
Triglycerides	1.062 (0.908, 1.242)	0.4546
HDL cholesterol	0.943 (0.792, 1.122)	0.5081
LDL cholesterol	0.968 (0.873, 1.073)	0.5385

## Discussion

Involving 11 VitC-related SNPs as IVs and genetic statistics from the ECAC (12,906 EC cases and 108,979 controls), we used a bi-directional MR method to investigate the putative causality between increased VitC concentrations and EC risk for the first time. MR results showed that genetically predicted per SD increase (~20 μmol/L) in VitC at physiological levels was causally associated with a 37% higher EC risk, independent of confounders and mediators.

Our MR finding was consistent with the only cohort study focusing on this issue, which included 221 EC cases, that intake of VitC was correlated with a slightly increased risk of EC (10%) while without statistical significance (25). Similarly, a case-control study that included 42 EC cases and 68 controls indicated a 13% higher EC risk with the increment of VitC intake (26). However, a study proposed by Negri et al., which contained 368 cases and 713 controls, demonstrated that the EC risk decreased by 40% in the highest quintile than the lowest quintile of VitC intake (27). In addition, the other two studies showed negative association (28, 29) and most other case-control studies indicated null (8, 25, 30–33) association. The latest dose-response meta-analysis enrolling one cohort study and nine case-control studies showed that per 50 mg increase in VitC intake decreased the risk of EC by 15% (7).

Nevertheless, since traditional observational studies are susceptible to potential confounders or reverse causality, limitations of previous studies existed. First, the number of EC cases enrolled in previous studies was rather small, with the maximum sample size up to 368, which may lack substantial statistical performance to assess the causal effect of increased VitC concentrations on EC. Second, given EC is comprised of a distinct set of histological subtypes and differences in histology related to differences in molecular features and clinical behaviors, no studies have managed to investigate the correlations between VitC concentrations and different histotypes of EC. Third, none of the previous studies managed to control BMI, while high BMI is regarded as a pivotal risk factor for EC. Similarly, the use of medication (e.g., oral contraceptive) or hormonal therapy can impact the incidence of EC, while no studies have taken these confounders into consideration. More importantly, no prospective large-scale cohort studies have been implemented currently. Thereby, it is insufficient to completely address the causal direction between increased VitC concentrations and EC.

Previous MR studies have investigated the effects of VitC levels with several health issues (e.g., Alzheimer's disease and hyperuricemia) with only one SNP as IV (34, 35). In the field of cancer, Fu et al. implemented the MR analysis to probe into the causal direction between VitC concentrations and lung, colon, rectal, prostate, and breast cancer with 11 SNPs as IVs while they found null associations (36). Our study first investigated the causal relationships between VitC and EC risk with bi-directional MR design, of which several advantages were listed as follows. First, we used the most comprehensive and proved GWASs-identified SNPs as IV sets. With large sample size (*n* = 52,018 for circulating VitC levels, *n* = 121,885 for EC) and strongly associated IVs (power = 100.0%), our MR study with substantial statistical performance may estimate the causal effect more precisely. Second, we attempted to address the three key assumptions underlying the MR design with robust methods (e.g., MR-PRSSO and excluding pleiotropic SNPs), resulting in mostly unbiased findings. Third, we evaluated the influence of potential confounders and mediators, including obesity, BMI, hypertension, LDL cholesterol levels, HDL cholesterol levels, triglycerides levels, and diabetes, on the VitC-EC pathway. Considering the gross effect that genetically predisposed increased VitC concentrations were causally correlated with an increased EC risk, these confounders and mediators were unlikely to influence the VitC-EC relation, implying a comparatively independent relationship between them.

In addition, several limitations should be noted. First, despite using the most comprehensive set of SNPs currently, it solely explained a small part of the variance of increased VitC concentrations in the population. It is probable that several unknown VitC-related SNPs may also influence the progression of EC. Second, the observed causal effect between genetically predisposed elevated concentrations of VitC and EC was modest, with a 37% higher risk of EC. Therefore, the clinical significance of increased concentrations of VitC in the progression of EC is relatively limited and whether people with increased VitC levels should be monitored continuously remains uncertain. Third, we cannot evaluate the potential non-linear relationships between VitC levels and EC risk. Additionally, given that the IVs of both the exposure and outcome phenotypes were of European descent, whether our findings can be extended to other ethnicities was uncertain. Consequently, our results should not be considered definitive.

Since EC comprises a genetically and histologically broad range of tumors, the different signaling pathways underlying increased VitC concentrations to different subtypes of EC risk could be complicated and affected by various factors, whereas related research was quite limited. Since the circulating VitC levels were seldomly measured in most observational studies, the preventative effects of VitC against cancers were based on dietary intake or supplementation. As is well known, VitC cannot be composited by the human body and has to be gained from the diet (e.g., fruit and vegetable). Hence, the VitC maybe just a biomarker of vegetables and fruits consumption, and the reported protective effects of VitC were likely to be biased by other factors, such as fibers and polyphenols in vegetables and fruits. In this case, it is difficult to disentangle the unconfounded VitC-EC relation in traditional studies. Herein, we used the MR design analysis to dissect the relationship between genetically predicted VitC at physiological levels and EC risk. Under the circumstance that high pharmacological levels of VitC through intravenous injection alone or combined with other drugs manifested promising outcomes on treating cancers, it is essential for public health to determine whether maintaining the high physiological levels of VitC contributes to cancer prevention. Consequently, combined previous reports with our findings, universal screening of the general population for hypovitaminosis and maintaining high physiological VitC levels should not be supposed to be a tactic for primary EC prevention at present. As we preliminarily identified VitC as a risk factor of EC overall, future experimental and longitudinal studies are warranted to verify our findings and elucidate the possible mechanisms. Subsequently, people with high circulating VitC levels may need to be screened routinely (e.g., hysteroscopy and transvaginal ultrasound) (37), which may lower the mortality rates of EC in the future. Moreover, with the emerging data from GWAS and epidemiological studies, precisely defining the high-risk populations of EC and refining risk classification may lower the disease burden of EC. Meanwhile, it is pivotal for future studies to recognize the changeable risk factors (e.g., diets and lifestyles) of EC and take preventative measures subsequently to lower the incidence of EC.

## Conclusions

The present bi-directional MR study indicated a causal risk role of higher circulating VitC at physiological levels on an increased risk of EC in European descent, which was independent of confounders and mediators. Future large-scale GWASs with individual-level data and more SNPs of VitC, which can be used to build genetic scores of VitC metabolic pathway, and experimental studies, are warranted to better understand the mechanisms from VitC concentrations to EC.

## Data Availability Statement

The original contributions presented in the study are included in the article/Supplementary Material, further inquiries can be directed to the corresponding author.

## Ethics Statement

The study protocol complied with the principles of the Declaration of Helsinki and was approved by the Institutional Review Board of the National Clinical Research Center for Respiratory Disease of the First Affiliated Hospital of Guangzhou Medical University.

## Author Contributions

HP and XW: conception, design, collection, assembly of data, data analysis, and interpretation. All authors provided study materials or patients, contributed to writing the manuscript, and approved the final version of the manuscript.

## Funding

This work was supported by Cultivation of Guangdong College Students' Scientific and Technological Innovation (Climbing Program Special Funds) (Grant Numbers: pdjh2020a0480 and pdjh2021a0407).

## Conflict of Interest

The authors declare that the research was conducted in the absence of any commercial or financial relationships that could be construed as a potential conflict of interest.

## Publisher's Note

All claims expressed in this article are solely those of the authors and do not necessarily represent those of their affiliated organizations, or those of the publisher, the editors and the reviewers. Any product that may be evaluated in this article, or claim that may be made by its manufacturer, is not guaranteed or endorsed by the publisher.
